# Association of the Red Cell Distribution Width With the Glycemic Index and Lipid Profile in Patients With Type 2 Diabetes Mellitus

**DOI:** 10.7759/cureus.42800

**Published:** 2023-08-01

**Authors:** Mamdouh Allahyani

**Affiliations:** 1 Department of Clinical Laboratory Sciences, College of Applied Medical Sciences, Taif University, Taif, SAU

**Keywords:** cbc, lipid profile, hba1c, rdw, t2dm

## Abstract

Background

The aim of this study was to assess the association between the red cell distribution width (RDW) and the hemoglobin A1C (HbA1c) and lipid profiles in patients with type 2 diabetes (T2DM).

Materials and methods

This case-control study included 130 individuals with T2DM disease who were admitted to the Diabetic Center in Taif, Saudi Arabia, between August and December 2022. The patients were divided into two groups: pre-diabetic (45 patients) and diabetic (85 patients). A total of 65 healthy people were included in the study as controls. The (HbA1c) level, lipid profile, and complete blood count (CBC) were determined for each participant, and differences in those parameters between the groups were evaluated using the one-way ANOVA test or Kruskal-Wallis test. The association between different parameters, including the RDW, was evaluated using the Pearson correlation coefficient.

Results

Both the pre-diabetic and diabetic patients were obese and had high concentrations of triglycerides, cholesterol, and low-density lipoprotein (LDL). None of the diabetic patients had anemia of any type. However, the RDW was higher in the diabetic group than in the healthy controls and a significant difference was detected. A positive correlation was detected between the RDW and the HbA1c levels and lipid profiles.

Discussion

The size of the red blood cells varied in patients with T2DM, as demonstrated by the high RDW values. The RDW showed a positive correlation with the glycemic index and with the lipid profile in patients with T2DM, suggesting that it is a useful prognostic marker for managing patients with T2DM.

## Introduction

Diabetes mellitus (DM) is an epidemic disease, with approximately 463 million persons diagnosed with it. Of those, 90% are patients with type 2 DM (T2DM). Current estimates indicate that 700 million cases of DM will be reported in 2045 [1]. DM develops due to insulin resistance, which ultimately leads to reduced insulin secretion [2]. DM has a number of associated complications, such as nephropathy, neuropathy, and cardiovascular disease [2].

The prevalence of DM is high in the Middle East and especially in Saudi Arabia [3], where the incidence of DM is presently 23.7% in the Saudi population [4]. The main triggers of DM are lifestyle factors, including diet, smoking, and physical activity. Obesity is a particularly significant risk factor for the development of DM [3], and obesity rates have increased in the last decades, even doubling in some countries since 1980 [5]. DM is a global concern, and much money is now spent on treating patients with DM [3]. Therefore, management of the disease and regular check-ups are crucial.

The health status of normal individuals and patients with various diseases is commonly monitored using the complete blood count (CBC) [6]. In patients with T2DM, the CBC can be used as a follow-up test, which will help in reducing complications associated with the disease [7]. Some CBC parameters have also been used as prognostic markers for T2DM [6]. One of these markers is the red cell distribution width (RDW), which measures the variability in the sizes of red blood cells (RBCs) [8].

The RDW can be used diagnostically in patients with T2DM and other illnesses (6), as patients with T2DM frequently show alterations in various hematological properties, including changes in the structure, metabolism, and function of blood cells [9]. These alterations can be caused by different factors, such as excessive levels of reactive oxygen species (ROS), leading eventually to oxidative stress and the dysfunction of RBCs [9]. White blood cells are also reported to be altered in patients with T2DM [10]. However, the results reported for altered hematological parameters in patients with T2DM have been inconsistent [9]. In Saudi Arabia, hematological data for patients with T2DM are limited. Thus, this study aimed to investigate an association between RDW and hemoglobin A1c (HbA1c) and lipid profiles in T2DM by comparing pre-diabetic patients, diabetic patients, and healthy controls in Saudi Arabia.

## Materials and methods

Participants and research design

This case-control study included 130 individuals with T2DM who were admitted to the Diabetic Center in Taif, Saudi Arabia, between August and December 2022. Based on their determined glycemic indexes, the patients were divided into two groups: pre-diabetic (45 patients) and diabetic (85 patients). Patients with HbA1c values of 5.7-6.4% were considered pre-diabetic, while patients with levels 6.5 and above were considered diabetic [11]. As a control group, 65 healthy people were included in the study.

Inclusion criteria

The control group included subjects and presented no clinical evidence of diabetes or obesity. For the diabetic groups, only patients with T2DM were selected for the study.

Exclusion criteria

Individuals with type 1 DM, and diabetic patients with complications, such as nephropathy and retinopathy were excluded from the study. Individuals were also excluded from the study if they had any type of anemia.

Data collection

Demographic and clinical data were retrieved from the patients’ medical records. This was performed after receiving approval from the research ethics committee at Taif University (No. 43-133). Informed consent was obtained from all participants. Data for HbA1c, parameters of lipid profiles (triglycerides, cholesterol, low-density lipoprotein (LDL), high-density lipoprotein (HDL), and CBC components (RBC count, hemoglobin, hematocrit, mean corpuscular hemoglobin concentration (MCHC), mean corpuscular volume (MCV), mean corpuscular hemoglobin (MCH), and RDW) were included in the study. HbA1c was analyzed via an analyzer (Bio-Rad, New York, USA), the lipid profile was measured using an Architect c16000 chemistry analyzer (Abbott, USA), and the CBC was performed using an automated Mindray BC-2800 analyzer (Shenzhen, China). 

Statistical analysis

Data were processed using GraphPad Prism software (version 6.04, La Jolla, CA, USA). Data normality was tested using the Shapiro-Wilk test. Then, normally distributed data were analyzed using one-way ANOVA, whereas non-normally distributed data were analyzed using the Kruskal-Wallis test. The Pearson correlation coefficient was used to evaluate associations among different parameters. Quantitative data were written as mean and standard deviation (SD). A p-value less than 0.05 was considered statistically significant.

## Results

Both groups with T2DM (the pre-diabetic and diabetic patients) were significantly older than the control individuals (p = 0.000) (Table 1). The glycemic index (HbA1c) was also significantly higher in both pre-diabetic and diabetic groups than in the control individuals (p = 0.001). Data showed that the pre-diabetic group was overweight according to the BMI (27.7 ± 0.4) and the diabetic patients were obese (31.2 ± 0.9) (p = 0.002). The lipid profiles showed significantly higher levels of cholesterol, triglycerides, and LDL in the pre-diabetic and diabetic patients than in the normal group (p < 0.0001, 0.001, and 0.001, respectively). Conversely, the HDL was significantly lower in both the pre-diabetic and diabetic patients than in the controls (p = 0.040). Full demographic data are detailed in Table 1.

**Table 1:** Demographic data and biochemical analysis HbA1C: hemoglobin A1C, BMI: body mass index, HDL: high-density lipoprotein, LDL: low-density lipoprotein, TG: triglyceride, SD: standard deviation

Parameter	Control group (n = 65)	Pre-diabetic group (n = 45)	Diabetic group (n = 85)	P value
Age (years) (mean ± SD)	48.0 ± 2.0	51.3 ± 7.2	69.3 ± 10.9	0.000
HbA1c (%) (mean ± SD)	4.7 ± 0.7	6.0 ± 1.4	9.5 ± 3.3	0.001
Weight (kg)	59.7 ± 3.2	68.1 ± 2.2	91.6 ± 3.9	< 0.0001
BMI	25.3 ± 1.3	27.7 ± 0.4	31.2 ± 0.9	0.002
Cholesterol (mg/dl)	119.7 ± 9.5	148. 7 ± 21.7	247.0 ± 7.7	< 0.0001
TG (mg/dl)	98.1 ± 10.5	139.3 ± 22.1	227.3 ± 39.9	0.001
LDL (mg/dl)	89.6 ± 5.1	99.3 ± 10.7	134. 1± 8.5	0.001
HDL (mg/dl)	50.1 ± 4.3	42.6 ± 6.1	38.3 ± 10.5	0.040

The CBC was performed to investigate the anemic status in the patients with T2DM and to detect any differences in the RBC indices in the pre-diabetic and diabetic conditions. The patients with T2DM were not anemic, and no significant differences were observed in RBC, hemoglobin, and hematocrit values for either the pre-diabetic group or the diabetic group in comparison to controls (p = 0.975, 0.299, and 0.246, respectively). The pre-diabetic and diabetic patients also had slightly lower MCV, MCH, and MCHC values, but the differences were not significant (p = 0.067, 0.175, and 0.393, respectively). The RDW value was significantly higher in the diabetic group compared with that of the controls (p = 0.002, Table 2). 

**Table 2:** Levels of various blood parameters in diabetic and control groups RBCs: red blood cells, MCV: mean corpuscular volume, MCH: mean corpuscular hemoglobin, MCHC: mean corpuscular hemoglobin concentration, RDW: red blood cell distribution width, SD: standard deviation

Parameter	Non-diabetic group (n = 65)	Pre-diabetic group (n = 45)	Diabetic group (n = 85)	P value
RBCs (mean ± SD) x 10^6 ^/ml	4.8 ± 0.4	4.5 ± 0.8	4.6 ± 0.9	0.975
Hemoglobin (g/dL) (mean ± SD)	14.1 ± 1.6	13.5 ± 2.5	13.3 ± 3.6	0.299
Hematocrit (%) (mean ± SD)	45.3 ± 3.5	42.0 ± 3.0	41.4 ± 2.1	0.246
MCV (fL) (mean ± SD)	86.1 ± 3.1	82.6 ± 4.5	81.6 ± 5.6	0.067
MCH (pg) (mean ± SD)	30.1 ± 1.9	28.2 ± 3.1	27.3 ± 4.5	0.175
MCHC (g/dL) (mean ± SD)	34.3 ± 2.1	33.1 ± 1.3	32.4 ± 2.9	0.393
RDW (%) (mean ± SD)	12.7 ± 0.6	13.5 ± 1.0	15.2 ± 4.6	0.002

**Figure 1:**
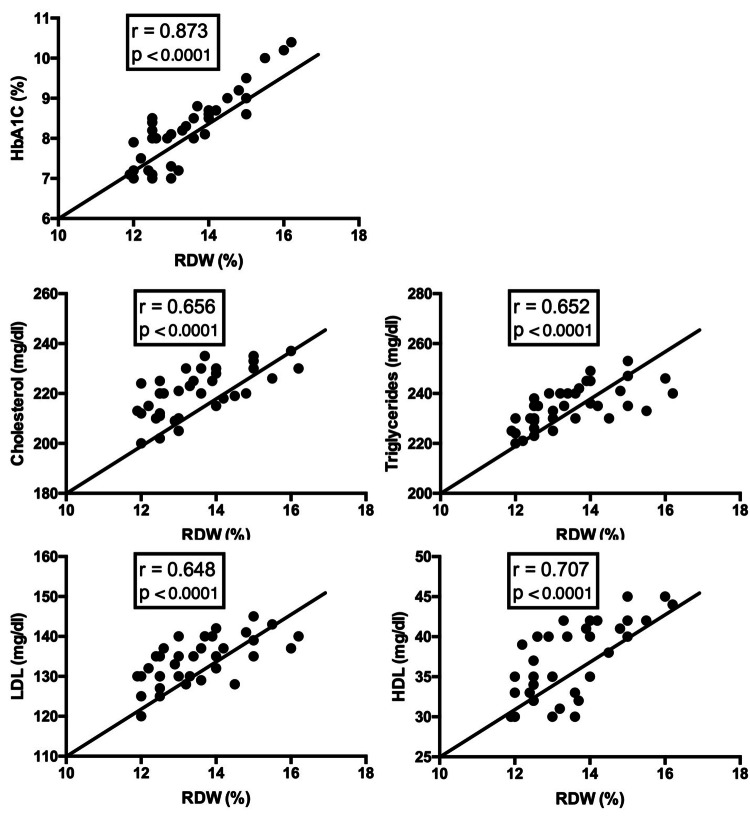
Correlation between the RDW and various parameters RDW and different values were plotted in Prism GraphPad software, and the correlation was calculated using Pearson’s coefficient analysis

Calculation of the correlation coefficients between the RDW and various parameters revealed a positive correlation between the RDW and HbA1c (p < 0.0001). A significant positive correlation was also detected in the percentage of RDW with cholesterol, triglyceride, LDL, and HDL values (all p < 0.0001, Figure 1).

## Discussion

DM is a global health issue, and the number of diagnosed cases is dramatically increasing worldwide [1]. Thus, the management and follow-up of this disease is of worldwide importance. Our aim in the present study was to evaluate an association between some CBC parameters and the HbA1c levels and lipid profiles in patients with T2DM (both pre-diabetic and diabetic patients). Both the pre-diabetic and diabetic patients were determined to be obese, but not anemic. However, the RDW percentage was significantly higher in the diabetic patients than in the healthy controls and showed a positive correlation with both the HbA1c level and the lipid profile in that group.

In the present study, the diabetic patients were obese, with high BMI values and high levels of cholesterol, triglycerides, and HDL. These findings are consistent with another study that showed obesity among the majority of patients diagnosed with T2DM [12]. Obesity is a well-known risk factor for DM, and lipid accumulation in patients with DM impairs insulin signaling and reduces glucose uptake [5]. 

Some parameters of the CBC, such as MCV, MCH, and MCHC, did not differ significantly when the diabetic patients were compared with the healthy control group. These findings indicated that T2DM patients were not anemic. Previous data regarding the presence of anemia in patients with T2DM have been inconsistent. Similar to our data, pre-diabetic and diabetic patients with T2DM disease have been reported to show normal values of RBCs, hemoglobin, hematocrit, MVC, MCH, and MCHC [13]. However, another study suggested that patients with T2DM were anemic and that the levels of MCV, MCH, and MCHC had no significant correlation with HbA1c [8].

Despite the absence of anemia in our patients, the RDW percentage was shown to be higher in the diabetic group than in the controls. One previous study has shown that an RDW increase has a strong correlation with HbA1c [8], in agreement with our findings. The RDW is recognized as an excellent prognostic tool for various diseases. A high RDW percentage indicates that the RBCs have different sizes (anisocytosis), and this is associated with ineffective erythropoiesis [13]. Increases in the RDW percentage have also been associated with cardiovascular disease [14], suggesting heart disease as a further health risk in our patients with diabetes. The diabetic group members in the present study were obese, and a strong association was found between the RDW and the lipid profile in the diabetic group. It was previously reported that RDW was strongly correlated with obesity [15].

Changes in some parameters of the CBC, especially the RDW, in patients with T2DM are also evident in some existing reports [14-17]. Spherocytosis has been observed in patients with T2DM and was strongly associated with hyperglycemia [14]. Alterations in the lipids and proteins found on the RBC membrane have also been reported in patients with T2DM and might contribute to increases in the RDW percentage [16]. Furthermore, alterations in the deformability of RBCs have been shown in patients with T2DM, with increased RBC aggregation, leading potentially to microcirculation issues in those patients [17]. Finally, the blood viscosity is increased, with reduced RBC mobility, in patients with T2DM [18]. 

A relatively small number of patients was enrolled in the current study; therefore, further studies with larger samples are recommended. A further recommendation is to compare the RDW percentages in T2DM patients who have different health complications.

## Conclusions

The RBC is one of the cells affected by T2DM and shows altered function, structure, and metabolism. The findings of the current study indicated a strong association between the RDW and both the glycemic index and the lipid profile in patients with T2DM. The RDW could therefore serve as a marker for the management of patients with this disease.
